# Inflammatory Breast Carcinoma: Elevated microRNA miR-181b-5p and Reduced miR-200b-3p, miR-200c-3p, and miR-203a-3p Expression as Potential Biomarkers with Diagnostic Value

**DOI:** 10.3390/biom10071059

**Published:** 2020-07-16

**Authors:** Sarah Atef Fahim, Mahmoud Salah Abdullah, Nancy A. Espinoza-Sánchez, Hebatallah Hassan, Ayman M. Ibrahim, Sarah Hamdy Ahmed, George Shakir, Mohamed A. Badawy, Nadia I. Zakhary, Burkhard Greve, Mohamed El-Shinawi, Martin Götte, Sherif Abdelaziz Ibrahim

**Affiliations:** 1 Biochemistry Program, Chemistry Department, Faculty of Science, Cairo University, Giza 12613, Egypt; sarahatef2013@gmail.com; 2 Biotechnology/Biomolecular Chemistry Program, Chemistry Department, Faculty of Science, Cairo University, Giza 12613, Egypt; mahmoudrete@icloud.com (M.S.A.); sarahhamdy622@yahoo.com (S.H.A.); george.shakir92@gmail.com (G.S.); 3 Department of Gynecology and Obstetrics, Münster University Hospital, 48149 Münster, Germany; latmw_yo@hotmail.com; 4 Department of Zoology, Faculty of Science, Cairo University, Giza 12613, Egypt; aheba@sci.cu.edu.eg (H.H.); ayman.maher@aswanheartcentre.com (A.M.I.); 5 Institute for Cardiovascular Prevention, Ludwig-Maximilians-University, 80539 Munich, Germany; 6 Chemistry Department, Faculty of Science, Cairo University, Giza 12613, Egypt; mabadawy52@gmail.com; 7 Cancer Biology Department, National Cancer Institute, Cairo University, Cairo 11796, Egypt; nadia.zakhary@nci.cu.edu.eg; 8 Department of Radiotherapy–Radiooncology, University Hospital Münster, 48149 Münster, Germany; greveb@uni-muenster.de; 9 Department of General Surgery, Faculty of Medicine, Ain Shams University, Cairo 11566, Egypt; mohamedshinawi@med.asu.edu.eg

**Keywords:** inflammatory breast cancer, microRNAs, miR-181b-5p, miR-200b-3p, miR-200c-3p, miR-203a-3p, miR-1-3p, *ZEB2*, hub genes

## Abstract

Inflammatory breast cancer (IBC) is a rare yet aggressive breast cancer variant, associated with a poor prognosis. The major challenge for IBC is misdiagnosis due to the lack of molecular biomarkers. We profiled dysregulated expression of microRNAs (miRNAs) in primary samples of IBC and non-IBC tumors using human breast cancer miRNA PCR array. We discovered that 28 miRNAs were dysregulated (10 were upregulated, while 18 were underexpressed) in IBC vs. non-IBC tumors. We identified 128 hub genes, which are putative targets of the differentially expressed miRNAs and modulate important cancer biological processes. Furthermore, our qPCR analysis independently verified a significantly upregulated expression of miR-181b-5p, whereas a significant downregulation of miR-200b-3p, miR-200c-3p, and miR-203a-3p was detected in IBC tumors. Receiver operating characteristic (ROC) curves implied that the four miRNAs individually had a diagnostic accuracy in discriminating patients with IBC from non-IBC and that miR-203a-3p had the highest diagnostic value with an AUC of 0.821. Interestingly, a combination of miR-181b-5p, miR-200b-3p, and miR-200c-3p robustly improved the diagnostic accuracy, with an area under the curve (AUC) of 0.897. Intriguingly, qPCR revealed that the expression of zinc finger E box-binding homeobox 2 (*ZEB2*) mRNA, the putative target of miR-200b-3p, miR-200c-3p, and miR-203a-3p, was upregulated in IBC tumors. Overall, this study identified a set of miRNAs serving as potential biomarkers with diagnostic relevance for IBC.

## 1. Introduction

Breast cancer is the most frequent cancer in women, with over two million new cases in 2018 [1]. It ranks as the fifth cause of cancer-related death, with 627,000 cases in 2018. Inflammatory breast cancer (IBC) is a rare but the most aggressive variant of breast cancer that has a rapid progression from the onset of disease [2]. It is clinically characterized by edema, redness, and dimpling of the skin (peau d’orange) caused by tumor emboli blocking dermal lymphatics [3]. Moreover, 55–85% of patients present with positive metastatic lymph node (LN) involvement and up to one-third of patients have distant metastasis at diagnosis [4]. IBC only accounts for 2–4% of all breast cancer cases, but it is responsible for 7–10% of breast cancer-related deaths [3,5]. IBC patients have poorer survival rates compared with non-IBC patients despite of the presence of multiple improved treatments [5]. In Egypt, IBC accounts for 11% of the diagnosed breast cancer cases and only 24% of IBC exhibit 4-year survival versus 67% of non-IBC, despite the similar multidisciplinary care given for both diseases [4,6]. Although several studies have been conducted to discover new diagnostic and predictive biomarkers [7,8,9,10], IBC is still misdiagnosed due to the lack of accurate and reliable molecular biomarkers.

MicroRNAs (miRNAs) are a class of small (19–22 nucleotides) endogenous noncoding highly conserved single-stranded RNAs [11]. miRNAs are known as mRNA regulators, as they negatively regulate the expression of numerous genes at the post-transcriptional level by forming base-pairing with target mRNAs, thereby facilitating mRNA degradation or translational silencing [11,12]. miRNA expression can be dysregulated under pathological conditions, including cancer, thus acting as tumor suppressors or oncogenes based on their regulation of the downstream mRNA/protein targets. Consequently, miRNAs play a pivotal role in each step of cancer development and progression, including cell differentiation, apoptosis, cell proliferation, invasion, metastasis, and angiogenesis via the modulation of a complex network of signaling pathways [13,14]. Several studies reported the diagnostic, predictive, and prognostic values for dysregulated miRNAs in breast cancer [15]. In three previous studies, miRNA signatures were profiled using different experimental approaches based on PCR and microarray for a cohort of IBC patients; however, a large extent of discrepancy in the identified miRNA signature was reported [9,16,17]. Therefore, a consensus for a miRNA signature for IBC is still fully unexplored. The aim of this study was to identify and verify the IBC-related miRNA signature, which may contribute to the pathogenesis of the disease and may act as useful diagnostic molecular biomarkers and therapeutic targets.

## 2. Materials and Methods

### 2.1. Primary Human Breast Tissue Samples

This study was approved by the institutional review board (IRB#00006379) of Ain Shams University hospital. Patients and controls enrolled in the present study signed an informed consent form to participate in this study in accordance with the Declaration of Helsinki. Carcinoma tissue specimens of 35 females comprising of 18 non-IBC and 17 IBC patients were collected after curative surgery at Ain Shams University hospital. Normal mammary tissues collected from healthy females, who underwent breast reduction mammoplasty surgery were used as controls (*n* = 5). Normal mammary tissues were from females with an age range of 39–49 years. As depicted in Table 1, we enrolled breast cancer patients with an age range of 32–82 years and subgrouped them into patients with non-IBC and IBC. A significantly higher involvement of ≥ 4 LNs (*p* = 0.02), and lymphovascular invasion (*p* = 0.005) was observed in IBC relative to non-IBC, reflecting typical characteristics of IBC [3]. Other clinic-pathological features, including age, tumor size, tumor grade, hormonal receptor status, and human epidermal growth factor receptor 2 (Her2), did not show any significant difference between both groups. The fresh tumor tissue samples were stored in RNA later (Sigma-Aldrich, Munich, Germany) at −80 °C until total RNA extraction. The clinic-pathological features of IBC and IBC patients enrolled for miRNA PCR array analysis are presented in Supplementary Table S1.

### 2.2. Total RNA Extraction and Human Breast Cancer miRNA PCR Array

Total RNA was extracted using QIAzol lysis reagent (Qiagen, Hilden, Germany) and the Direct-zol ^TM^ RNA kit (Zymo Research, CA, USA) according to the manufacturer’s instruction. Total RNA isolated from 9 tumor tissue samples of either IBC or non-IBC was pooled, and the RNA concentration and purity were assessed using Nanodrop (Tecan, Switzerland). Then, 250 ng of total RNA were converted into cDNA using the miScript RT II kit (Qiagen) according to the manufacturer’s instructions. miRNAs profiling was conducted using human breast cancer miRNA PCR Array-MIHS-109Z (Qiagen). All reactions were performed using SYBR Green-based real-time PCR with the miScript SYBR Green PCR Kit (Qiagen) with 1 µL of cDNA in a 25-µL reaction per well in a Step One Plus Real Time-PCR Detection System (Applied Biosystems, CA, USA). The miScript cycling conditions were as follows: An initial activation at 95 °C for 15 min, followed by 40 cycles of 94 °C for 15 s, at 55 °C for 30 s, and 70 °C for 30 s. Data were analyzed using the miScript miRNA PCR Array web-based software (http://pcrdataanalysis.sabiosciences.com/mirna/arrayanalysis.php). All Ct values greater than 35 were excluded. The mean of the cycle threshold (Ct) of cel-miR-39, the small nuclear RNA SNORD61, SNORD68, SNORD72, SNORD95, SNORD96A, and RNU-6 was used as the endogenous control. ΔΔCt for each miRNA was calculated using the formula: ΔΔCt = ΔCt (IBC) − ΔCt (non-IBC), where non-IBC is the control sample and IBC is the experimental sample. For cell lines, microRNA isolation was performed using the innuPREP RNA Mini Kit (Analytik Jena AG, Jena, Germany) according to the manufacturer’s instructions.

### 2.3. Quantitative Real-Time PCR

The expression of five selected dysregulated miRNAs as identified by miRNA PCR array was validated in an independent patient cohort by qPCR to evaluate their potential diagnostic value to differentiate between patients with non-IBC and IBC. The expression levels of miRNAs in breast carcinoma tissues of 18 non-IBC and 17 IBC patients and 5 normal breast tissues from healthy females were detected using the miScript SYBR Green PCR Kit (Qiagen) and miScript primer Assays (Qiagen) for Hs_miR 203a (MS00003766), Hs_miR 200b (MS00009016), Hs_miR 200c (MS00003752), Hs_miR 181b (MS00006699), and Hs_miR-1 (MS00008358). The miScript cycling conditions were as follows: An initial activation at 95 °C for 15 min, followed by 40 cycles of 94 °C for 15 s, at 55 °C for 30 s, and at 70 °C for 30 s. Small nucleolar RNA (SNORD68, MS00033712) was used as the endogenous control. Fold change for each miRNA was expressed as 2^−ΔΔCT^ and log2 transformed after normalization to the endogenous control. For *ZEB2* mRNA expression-level detection, total RNA isolated from primary normal breast tissues or breast carcinoma tissues was reverse transcribed into cDNA using the High-Capacity cDNA Reverse Transcription Kit (Thermo scientific, ON, Canada) and qPCR of *ZEB2* mRNA expression was performed using Brilliant SYBR Green qPCR master mix (Applied Biosystems, CA, USA). The following primers were used: *ZEB2* primer (upstream 5′-TGGGCTAGTAGGCTGTGTCCA-3′ and downstream 5′-TCATCTTCAACCCTGAAACAGAGG-3′) and the endogenous control for normalization glyceraldehyde3-phosphate dehydrogenase (*GAPDH*, #QT00079247, Qiagen). MicroRNAs isolated from cell lines were converted into cDNA using the TaqMan MicroRNA Reverse Transcription kit (Applied Biosystems Inc., Foster City, CA, USA). The following TaqMan probes (all Applied Biosystems) were used for real-time PCR: 002222 (hsa-miR-1-3p), 002251 (hsa-miR-200b), and 001093 (RNU6B, housekeeping control).

### 2.4. Cell Culture

The human non-IBC cell lines MCF-7 (luminal A) and MDA-MB-231 (triple-negative) were purchased from ATCC/LGC Promochem (Wesel, Germany). The IBC SUM149 (triple-negative) cell line was a gift from Prof. Dr. Robert J. Schenider (School of Medicine, New York University, New York, NY, USA). MCF-7 and MDA-MB-231 cells were cultured in RPMI and DMEM with 1% glutamine, 1% penicillin/streptomycin antibiotic mixture, and supplemented with 10% fetal calf serum (FCS) (Biochrom GmbH, Berlin, Germany), and maintained in a humidified atmosphere of 5% and 7.5% CO_2_ at 37 °C, respectively. SUM149 cells were cultured in HAM’s-F12 containing 1% penicillin/streptomycin, 1% glutamine, and supplemented with 5% fetal calf serum (FCS), 1 µg/mL hydrocortisone, and 5 µg/mL insulin. SUM149 cells were maintained in a humidified atmosphere of 5% CO_2_ at 37 °C. All reagents were purchased from Sigma-Aldrich Chemie (Taufkirchen, Germany). Total cell lysates of the aforementioned cell lines were used to extract miRNAs and perform Taqman probe-based real-time PCR as described above.

### 2.5. Prediction of miRNA Target Genes and GO Function and KEGG Pathway Analysis

We searched for the target genes of the up- and downregulated miRNAs in the miRbase database (miRDB) (http://mirdb.org/index.html) [18,19]. We selected as differentially expressed genes (DEGs) for the functional and network analyses, all the target genes with a score > 95. To analyze which biological process (BP), molecular function (MF), cellular component (CC), and pathways might be affected by the down- and upregulated miRNAs, the online Database for Annotation, Visualization and Integrated Discovery (DAVID) software was employed. DAVID software uses well-known classification systems, including Gene Ontology (GO), Kyoto Encyclopedia of Genes and Genomes (KEGG), and BioCarta path (https://david.ncifcrf.gov/home.jsp) [20,21]. A false discovery rate (FDR) < 0.05 was chosen as the cut-off criterion.

### 2.6. Integration of the PPI Network and Identification of Significant Candidate Genes (Hub Genes) and Pathways

Using the online platform STRING (https://string-db.org), which uses three different databases: GO, Protein families (Pfam), and KEGG to predict protein-protein interaction (PPI) networks [22], we developed a single network of all DEGs, including the candidate genes targeted by differentially up- and downregulated miRNAs. The PPI network was exported to Cytoscape software (version 3.8.0) [23] for further network analyses. We used the Cytoscape plugin Molecular Complex Detection (MCODE) to identify the most important clusters or modules from the densely/highly interconnected regions within the network. The four most significative modules, containing the hub genes, were selected by the node score cutoff = 0.2, k-core = 2, max. depth from seed = 100, and degree cutoff = 2. We selected the modules with a score > 10. The hub genes were mapped into String to perform an enrichment pathway analysis. FDR is the parameter computed by string for all GO and enrichment pathways.

### 2.7. Kaplan-Meier Plots and Survival Analysis

In order to further characterize the clinicopathological relevance of the miRNAs identified in our study, we analyzed their impact on the survival of breast cancer patients. As no data were available for the rare entity of IBC, we extended our analysis to a patient collective composed of all major subtypes of breast cancer. The METABRIC dataset (number of patients *n* = 1262) was employed in this analysis via Kaplan-Meier (KM) plotter, an online platform combining gene microarray data and patient overall survival rates [24] Patients were divided using an auto-selection feature based on the median and quartile expression levels of the studied miRNAs and quality controlled for redundant samples and biased assays. Median survival was reported in months and compared for significance with a hazard ratio and *p*-value generated on the graph. A *p*-value of < 0.05 was considered statistically significant (Log-rank, Chi-squared test). The overall survival status was analyzed either without patients filtering, or with a further categorization of patients based on molecular subtypes (luminal A, luminal B, Her-2, and triple-negative), tumor grade (grade I, II, and III), and LN status (positive and negative).

### 2.8. Statistical Analysis

Data were analyzed using IBM SPSS advanced statistics version 20 (SPSS Inc., Chicago, IL, USA). Numerical data were expressed as the mean and standard deviation (SD) or standard error mean (SEM), unless otherwise stated. Chi-square test or Fisher’s exact test was used to examine the relation between qualitative variables. For comparison between 2 groups, we used Mann-Whitney U-test or unpaired Student’s *t*-test. The receiver operating characteristic (ROC) curve was used for the diagnostic performance of miRNAs to distinguish between IBC and non-IBC groups. The area under the curve analysis and the Youden index were applied to determine the optimal cut-off point, sensitivity, and specificity of each miRNA using MedCalc Software version 19.2.0. A *p*-value < 0.05 was considered significant.

## 3. Results

### 3.1. A Subset of miRNAs Is Differentially Expressed in IBC Tumors

**Figure 1:**
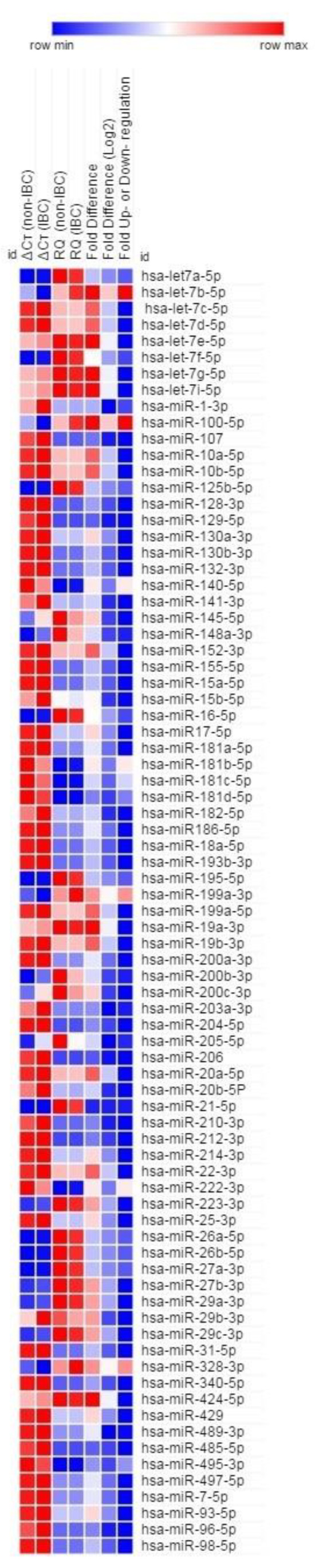
Aberrant expression of microRNA (miRNA) patterns in primary carcinoma tissue specimens of inflammatory breast cancer (IBC) vs. non-IBC. Assisted by the miScript miRNA PCR array, the expression levels of 84 miRNAs were profiled. The heat map shows 79 differentially expressed miRNAs with a Ct less than 35 for 9 pooled sample of IBC as opposed to 9 pooled samples of non-IBC. Data are expressed as log2 and were analyzed, and the heat map was generated using web-based software (http://pcrdataanalysis.sabiosciences.com/mirna/arrayanalysis.php) and Morpheus.

To screen differentially expressed miRNAs in tumor tissues of IBC vs. non-IBC, we employed the human breast cancer miRNA PCR array to generate a discovery dataset. Our array data showed that out of 84 profiled miRNAs, 28 miRNAs were dysregulated (10 were upregulated by approximately 1.7-fold, while 18 were underexpressed by less than 0.5-fold) in IBC tumors vs. non-IBC as depicted in Figure 1 and Table 2.

Importantly, among the downregulated miRNAs were miR-200 family members (miR-200b-3p, miR-200c-3p, and miR-141-3p), and the top downregulated miRNAs were miR-1-3p (0.1-fold), miR-203a-3p (0.2-fold), and miR-205-5p (0.2 fold) in IBC tumors vs. non-IBC.

### 3.2. Prediction of miRNA Target Genes and Enrichment Analyses

We used the miRDB online tool to find the potential target genes from differentially expressed miRNAs. All the target genes with a score > 95 were selected. The analysis showed 657 DEGs as target genes for upregulated and 1224 DEGs for downregulated miRNAs. Then, the functions and pathway enrichment of the selected miRNA target genes, including the candidate genes targeted by differentially upregulated and downregulated miRNAs (a total of 1881), were analyzed on the DAVID website. GO analysis allows target genes of differential miRNA expression to be classified into the BP, CC, and MF. Table 3 shows the five most significant enrichment terms (*p* < 0.05) in each category.

DEGs of upregulated miRNAs were mainly enriched in the categories of regulation of transcription, regulation of RNA metabolic process, and synapse organization, in the BP group. In the CC group, enriched terms were non-membrane-bounded organelle and vesicle, apical part of cell, and elongation complex, while in the molecular function group, the ion-binding category was prominent. Supplementary Material Table 1 shows the complete GO analysis for DEGs of upregulated miRNAs. DEGs of downregulated miRNAs were associated with heart development, regulation of transcription, and protein amino acid phosphorylation in the BP group. In the CC group, the categories of nuclear lumen, Golgi apparatus, collagen, and endomembrane system were enriched. Finally, in the MF, DEGs were associated with protein kinase, transcription factor, and transcription repressor activity (Table 3). Supplementary Material Table 2 shows the complete GO analysis for DEGs of downregulated miRNAs. According to the KEGG pathway enrichment analysis, DEGs of upregulated miRNAs were mainly enriched in extracellular matrix (ECM)-receptor interactions, pathways in cancer, melanoma, glioma, and the tumor suppressor protein p53 pathway, whereas DEGs of downregulated miRNAs were enriched in the categories of axon guidance, mitoegn activated protein kinase (MAPK) signaling, focal adhesion, and prostate cancer (Table 4, Supplementary Material Tables 1 and 2).

### 3.3. Identification of Hub Genes and Enrichment Pathways from DEG PPI Networks

**Figure 2:**
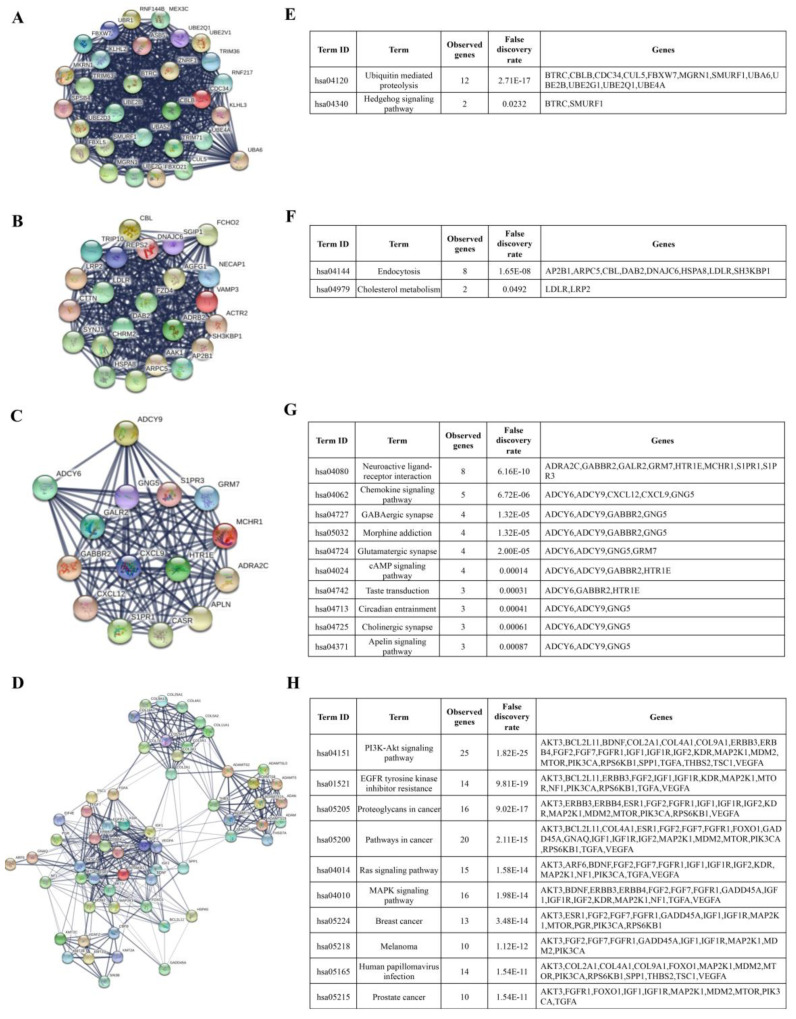
Hub genes protein-protein interactions (PPI) and enrichment pathways. Using the String database, we analyzed the hub genes present in most significant modules obtained from the Cytoscape plugin Molecular Complex Detection (MCODE) analysis. (**A**–**D**) shows the protein–protein interaction network of 30 hub genes present in module A, 23 in module B, 15 in module C, and 60 in module D. (**E**–**H**) the enrichment pathway of the hub genes function present in each module. < 1.0e-16 is the *p*-value for the entire PPI network of each module. The false discovery rate (FDR) is the parameter computed by String for each enrichment pathway for each module.

To identify the hub genes, we first had to upload the DEGs including the candidate genes targeted by differentially up- and downregulated miRNAs (a total of 1881) to the String database, choosing a medium confidence score > 0.4 to construct the PPI. Figure S1 shows an immense network where most of the genes are highly interconnected. Then, the results of the network (as.txt) were imported to Cytoscape to find the hub genes. For this purpose, we chose the MCODE plug in Cytoscape, which yielded a result of 35 clusters (Supplementary Material Table 3). All these clusters or modules are present in the immense PPI network in Figure S1. Then, we selected the top four significative modules with a score > 10 in which the hub genes were found. From a total of 1881 uploaded genes, only 128 represent the hub genes: 30 genes for module 1, 23 genes for module 2, 15 genes for module 3, and 60 genes for module 4 (Supplementary Material Table 3). Since each module represents molecules that have more interactions within themselves and fewer with the rest of the network and participate in specific cellular processes [25], the next step was to know the enriched pathways associated with these hub genes. For this purpose, all the genes of each module were taken, respectively, and were uploaded to the String platform. The hub genes are illustrated in the networks of Figure 2A–D. Figure 2A–D shows the PPI network of each module and as expected we could observe that the genes of each modules are highly interconnected, forming a single cluster. Only the genes of module 4 (Figure 2D), in addition to being interconnected, form more than one cluster. Enrichment analysis shows all the pathways in which the hub genes of each module were associated (Supplementary Material Table 4). We selected the 10 most significant enrichments to be represented (Figure 2E–H). We observed that hub genes in module 1 were associated with ubiquitin-mediated proteolysis and the Hedgehog signaling pathway (Figure 2E). Hub genes in module 2 were involved in endocytosis and cholesterol metabolism (Figure 2F). Hub genes in module 3 were involved in neuroactive ligand-receptor interaction, chemokine, 3′,5′-cyclic adenosine monophosphate (cAMP), and Apelin signaling pathways, GABAergic, glutamatergic, and cholinergic synapse, taste transduction, and circadian entrainment (Figure 2G), while hub genes in module 4 were more associated to cancer including breast, prostate cancer, and melanoma, the role of proteoglycans, signaling pathways, including phosphatidylinositol-3-kinase (PI3K/Akt), Ras, and MAPK, resistance to epidermal growth factor receptor (EGFR) inhibitors, and viral infection (Figure 2H).

In this silico analysis, GO showed that DEGs of upregulated miRNAs were mainly associated with regulation of transcription, while DEGs of downregulated miRNAs were associated with negative regulation of transcription, heart development, and amino acid phosphorylation.

### 3.4. Validation of Subsets of Candidate miRNAs in Carcinoma Tissue of IBC vs. Non-IBC

We next verified the expression of a subset of the miRNAs, miR-181b-5p, miR-200b-3p, miR-200c-3p, miR-203a-3p, and miR-1-3p, based on their fold expression and their role in breast cancer progression, especially whose expression may be related with features of IBC.

#### 3.4.1. Elevated miR-181b-5p Expression in IBC

**Figure 3:**
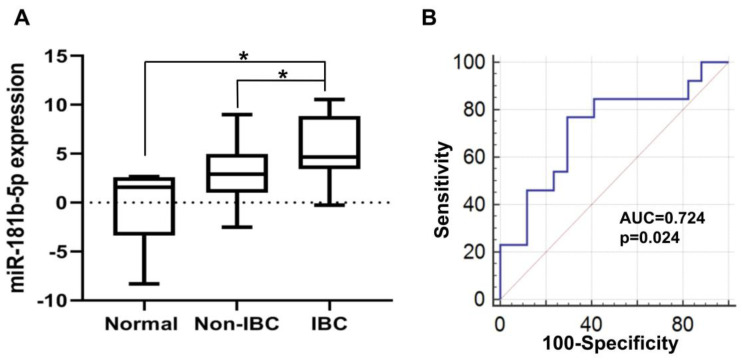
Expression and diagnostic value of miR-181b-5p in carcinoma tissues of non-IBC and IBC in comparison with normal breast tissue samples. (**A**) miR-181b-5p expression level is significantly elevated in carcinoma tissues of IBC (*n* = 13) relative to normal tissues (*n* = 5) and non-IBC (*n* = 17) as determined by qPCR. miR-181b-5p expression is log2-transformed and normalized to values of normal breast tissues collected during reduction mammoplasty. * *p* < 0.05 as determined by Mann-Whitney U-test. (**B**) Receiver operating characteristic (ROC) curve generated using expression levels of miR-181b-5p to discriminate between patients with non-IBC and IBC.

Our qPCR analysis revealed a significant upregulation of miR-181b-5p in carcinoma tissues of IBC as opposed to non-IBC patients and normal breast tissues (Figure 3A, both *p* < 0.05). Although expression of miR-181b-5p was upregulated in carcinoma tissues of non-IBC relative to normal tissues, it did not reach the significance level (Figure 3A). Moreover, miR-181b-5p had an AUC of 0.724 with a 77% sensitivity and 70.5% specificity for differentiating IBC from non-IBC patients as evaluated by ROC analysis (Figure 3B, *p* < 0.05).

#### 3.4.2. Low miR-200b-3p and miR-200c-3p Expression in IBC

**Figure 4:**
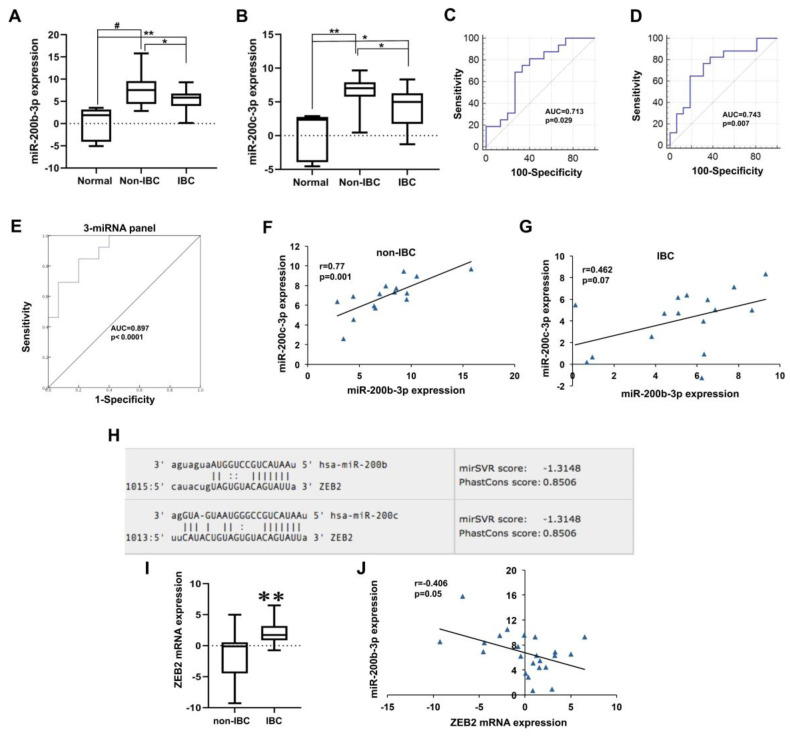
Expression of miR-200b-3p, miR-200c-3p, and their downstream target *ZEB2* mRNA in carcinoma tissues of non-IBC and IBC in comparison with normal breast tissue samples. (**A**) miR-200b-3p and (**B**) miR-200c-3p expression levels are significantly repressed in carcinoma tissues of IBC relative to non-IBC as determined by qPCR, whereas their expression levels in non-IBC and IBC are elevated when compared to normal breast tissue samples. miR-200b-3p and miR-200c-3p expression are log2-transformed and normalized to values of normal tissues collected during reduction mammoplasty. # * p* < 0.001, ** *p* < 0.01 and * *p* < 0.05 as determined by Mann-Whitney U-test. (**C**,**D**) ROC curves generated using expression levels of miR-200b-3p and miR-200c-3p to discriminate between patients with non-IBC and IBC, respectively. (**E**) ROC curves generated using a combination of expression levels of miR-181b-5p, miR-200b-3p, and miR-200c-3p as a miRNA panel to discriminate between patients with non-IBC and IBC. (**F**,**G**) Spearman’s correlation between miR-200b-3p and miR-200c-3p expression in carcinoma tissues of non-IBC and IBC, respectively. (**H**) Predicted target sites of the zinc finger E box-binding homeobox 2 (*ZEB2*) 3′UTR aligned with the seed sequence of miR-200b-3p and miR-200c retrieved from the microRNA.org database. (**I**) The expression of *ZEB2* mRNA levels is upregulated in carcinoma tissues of IBC vs. non-IBC. ** *p* < 0.01 as determined by Mann-Whitney U-test. (**J**) An inverse association between *ZEB2* and miR-200b-3p expression in breast cancer tumors as determined by Spearman’s correlation.

Unlike miR-181b-5p, compared to non-IBC patients, carcinoma tissues of IBC exhibited significantly reduced levels of miR-200b-3p and miR-200c-3p by 31% and 33%, respectively, as determined by qPCR (Figure 4A,B, both *p* < 0.05). However, miR-200b-3p and miR-200c-3p were significantly overexpressed in both non-IBC (Figure 4A,B, *p* < 0.001 and *p* < 0.01, respectively) and IBC (Figure 4A,B, *p* < 0.01 and *p* < 0.05, respectively) when compared to normal breast tissue samples. miR-200b-3p and miR-200c-3p had an AUC of 0.713 and 0.743 with an 81% and 82% sensitivity and 60% and 63% specificity for discriminating IBC from non-IBC patients, respectively, as evaluated by ROC analysis (Figure 4C,D, *p* < 0.05 for miR-200b-3p, and *p* < 0.01 for miR-200c-3p). Interestingly, when we assessed the combinations of three miRNAs (miR-181b-5p, miR-200b-3p, and miR-200c-3p), the accuracy of discrimination IBC from non-IBC was improved with an AUC of 0.897 (Figure 4E, *p* < 0.0001), sensitivity of 84.6%, and specificity of 80% compared to the corresponding individual miRNAs as determined by ROC analysis.

Together, this suggests that changes in the expression of miR-200b-3p and miR-200c-3p from high levels in non-IBC to low levels in IBC may have an influence on epithelial-to-mesenchymal transition (EMT) and mesenchymal-to-epithelial transition (MET) phenotypes of the tumor entity, and in turn its pathogenesis and progression. This can be further influenced by the regulation of expression levels of their direct downstream targets. As expected, we observed a significant positive correlation between the expression of miR-200b-3p and miR-200c-3p in non-IBC patients (Figure 4F, r = 0.77, *p* < 0.01), and a trend for the same positive correlation in IBC patients (Figure 4G, r = 0.46, *p* = 0.07), as they belong to the same miR-200 family. As illustrated in Figure 4H, the 3′UTR of *ZEB2* is a putative target gene for miR-200b-3p and miR-200c-3p, as generated by the miRanda algorithm using the online database [26]. Several reports discovered multiple direct targets for miR-200b and miR-200c, comprising the mesenchymal markers, including ZEB1/2 [27,28]. To this end, we examined *ZEB2* expression as a target for miR-200b-3p and miR-200c-3p in both patient groups. Consistently, our qPCR data indicate that IBC tumors displayed significantly higher mRNA levels of *ZEB2* by 3.8-fold compared with non-IBC (Figure 4I, *p* < 0.01). Although we found an inverse correlation between *ZEB2* and both miR-200b-3p and miR-200c-3p in non-IBC but not in IBC (data not shown), it did not reach the significance level. However, we found this inverse association between *ZEB2* and miR-200b-3p (Figure 4J, r = −0.406, *p* = 0.05) when the whole cohort of breast cancer patients was combined as one group regardless of IBC and non-IBC subtyping.

#### 3.4.3. Low miR-203a-3p Expression in IBC

**Figure 5:**
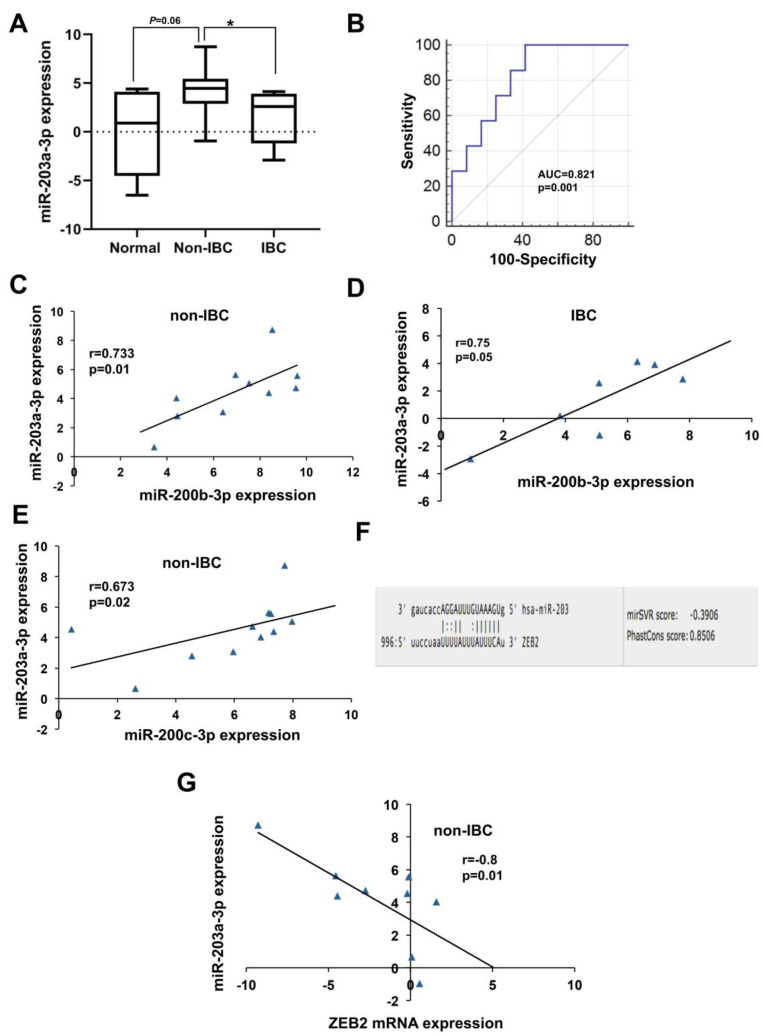
Expression and diagnostic value of miR-203a-3p in carcinoma tissues of non-IBC and IBC in comparison with normal breast tissue samples. (**A**) miR-203a-3p expression levels are significantly repressed in carcinoma tissues of IBC (*n* = 7) relative to non-IBC (*n* = 12) as determined by qPCR. miR-203a-3p expression is log2-transformed and normalized to values of normal tissues (*n* = 5) collected during reduction mammoplasty. * *p* < 0.05 as determined by Mann-Whitney U-test. (**B**) ROC curve generated using expression levels of miR-203a-3p to discriminate between patients with non-IBC and IBC. (**C**,**D**) Spearman’s correlation between expression of miR-203a-3p and miR-200b-3p in carcinoma tissues of non-IBC and IBC, respectively. (**E**) Spearman’s correlation between expression of miR-203a-3p and miR-200c-3p in carcinoma tissues of non-IBC. (**F**) Predicted target sites of the *ZEB2* 3′UTR aligned with the seed sequence of miR-203a-3p retrieved from the microRNA.org database. (**G**) Spearman’s correlation between expression of miR-203a-3p and *ZEB2* mRNA in tissues of non-IBC.

Relative to non-IBC, carcinoma tissues of IBC exhibited significantly reduced levels of miR-203a-3p by approximately 66% as determined by qPCR (Figure 5A, *p* < 0.05). miR-203a-3p expression was elevated, with a trend of significance (*p* = 0.06) in carcinoma tissues of non-IBC relative to normal breast tissue samples (Figure 5A). However, we did not observe an alteration in miR-203a-3p expression levels between carcinoma tissues of IBC and normal mammary tissues (Figure 5A). Of note, 59% (10 out of 17) of IBC samples had undetected miR-203a-3p expression versus only 33% (6 out of 18) of non-IBC samples. This suggests that loss or reduced expression of miR-203-3p can be used as a tissue biomarker to identify a cohort of IBC patients. More importantly, miR-203a-3p had an AUC of 0.821 (*p* < 0.01) with 100% sensitivity and 58.3% specificity for differentiating IBC from non-IBC patients as evaluated by ROC analysis (Figure 5B). Interestingly, we found a positive correlation between miR-200b-3p and miR-203a-3p in carcinoma tissues of non-IBC patients (Figure 5C; r = 0.73, *p* < 0.05) and of IBC (Figure 5D; r = 0.75, *p* = 0.05), and between miR-200c-3p and miR-203a-3p only in non-IBC patients (Figure 5E; r = 0.67, *p* < 0.05), suggesting a possible similar regulatory mechanism and biological functions. The 3′UTR of *ZEB2* is a candidate target gene for miR-203-3p (Figure 5F) using the online database according to the miRanda algorithm [26]. A significant inverse correlation between miR-203a-3p and *ZEB2* was detected only in carcinoma tissues of non-IBC (Figure 5G; r = −0.8, *p* < 0.05).

#### 3.4.4. miR-1-3p Expression in IBC

**Figure 6:**
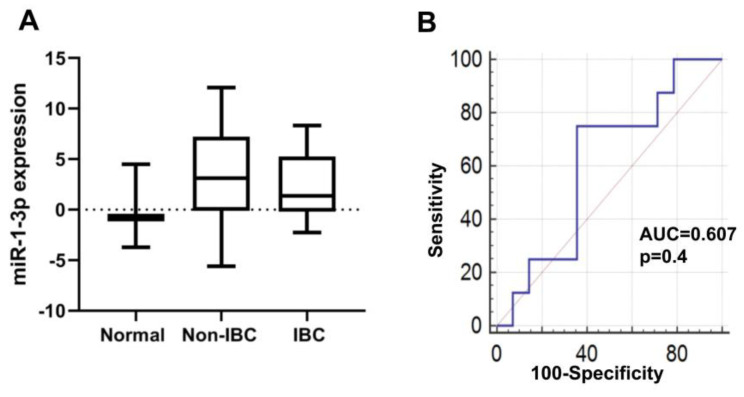
Expression and diagnostic value of miR-1-3p in carcinoma tissues of non-IBC and IBC in comparison with normal breast tissue samples. (**A**) miR-1-3p expression level is not altered in carcinoma tissues of IBC (*n* = 8) relative to normal breast tissues (*n* = 3) and non-IBC (*n* = 14) as determined by qPCR. miR-1-3p expression is log2-transformed and normalized to values of normal tissues collected during reduction mammoplasty. (**B**) ROC curve generated using expression levels of miR-1-3p to discriminate between patients with non-IBC and IBC.

Expression of miR-1-3p was diminished in the IBC tumor when compared to non-IBC tumor as determined by qPCR (Figure 6A); however, it did not reach the significance level. miR-1-3p expression was not significantly altered in carcinoma tissues of IBC and non-IBC relative to normal mammary tissue specimen. miR-1-3p had an AUC of 0.61 with a 75% sensitivity and 64% specificity for discriminating IBC from non-IBC patients, respectively, as evaluated by ROC analysis (Figure 6B), though it did not reach statistical significance. Importantly, 53% (9 out of 17) of IBC samples had undetected miR-1-3p expression versus only 22% (4 out of 18) of non-IBC samples with a significance trend (Table 5; *p* = 0.06). This may suggest that loss of miR-1-3p expression may be beneficial to identify a subset of IBC patients.

### 3.5. Expression of miR-1-3p and miR-200b in Non-IBC and IBC Cell Lines

**Figure 7:**
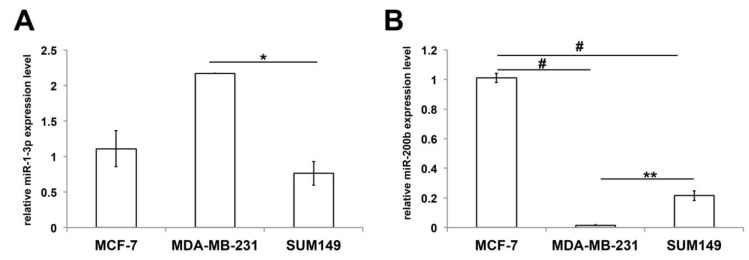
Expression levels of miR-1-3p and miR-200b in the non-IBC (MCF-7 and MDA-MB-231) and the IBC (SUM149) cells. qPCR was used to quantify the expression levels of miR-1-3p (**A**) and miR-200b (**B**) in different human breast cancer cell lines. Data represent the mean ± SEM, *n* = 3. * *p* < 0.05, ** *p* < 0.01, and # *p* < 0.001 as determined by Student’s *t*-test.

We next assessed the expression levels of miR-1-3p and miR-200b in the human non-IBC MCF-7 (ER+, luminal), MDA-MB-231 (triple-negative), and the IBC SUM149 (triple-negative) cell lines. Our qPCR data showed that miR-1-3p was significantly downregulated in SUM149 relative to MDA-MB-231 cells (*p* < 0.05) (Figure 7A). The expression of miR-200b was significantly higher in MCF-7 than in MDA-MB-231 (*p* < 0.001) and SUM149 cells (*p* < 0.001). However, the expression levels of miR-200b were elevated in SUM149 in comparison to MDA-MB-231 cells (*p* < 0.01) (Figure 7B).

### 3.6. Overall Survival Status of Breast Cancer Patients with Low and High Levels of the Identified miRNAs

We used KM plotter to study the association of the aforementioned identified miRNAs with overall survival, and to investigate the clinical relevance and prognostic value of their expression levels in breast cancer patients (miRNA module) [29]. KM plotter contains 41 previously published survival-associated miRNAs. We employed the METABRIC dataset, as it contains a considerable number of breast cancer patients (*n* = 1262), with various breast cancer molecular subtypes and follow-up data for up to 25 years. First, we stratified the patients according to the most common classifications, including the estrogen receptor (ER) and Her2 status. The overall survival of these breast cancer patients was analyzed based on the expression of up- and downregulated miRNAs. Table 6 shows that most of the upregulated miRNAs were associated with better overall survival in patients with ER-positive and Her2-negative tumors except miR-181b and miR-222, which were associated with worse overall survival in both classifications. Upregulation of let-7b, miR-100, and miR181d also correlated with good prognosis in Her2-positive tumors, while miR-181d was associated with worse survival in ER-negative tumors.

Of the downregulated miRNAs, miR-1, miR-148a, miR-205, miR-29b, miR-485, and miR-96 were associated with a better outcome in both ER-positive and Her2-negative tumors, while miR-129 correlated with worse survival in ER-positive and Her2-negative tumors. Interestingly, miR-141, miR-200c, and miR-210 were associated with poor survival in patients with both ER-positive and -negative and Her2-negative tumors. We did not find a positive correlation for the same miRNAs even with a high hazard ratio (HR) in Her2-positive tumors. miR-182 and miR-200b were associated with worse survival in patients with ER-negative tumors, whereas miR-15b and miR-206 correlated with poor outcome in Her2-negative tumors (Table 6).

**Figure 8:**
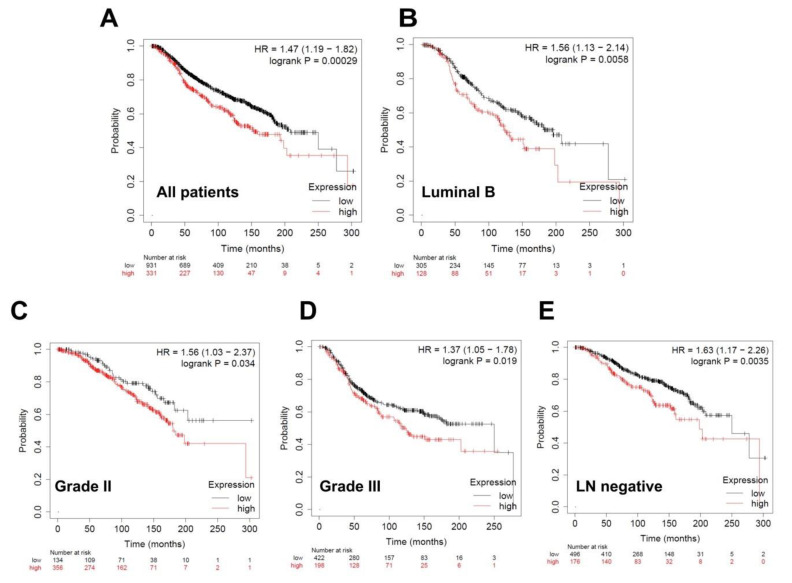
Overall survival curves according to miR-181b expression in breast cancer patients. High miR-181b expression correlated with worse outcome. Kaplan–Meier curves are plotted for expression of miR-181b in (**A**) all patients (*n* = 1262), (**B**) luminal B patients (*n* = 433), (**C**) patients with grade II (*n* = 383) and (**D**) patients with grade III (*n* = 620), and (**E**) LN-negative patients (*n* = 672). Curves were analyzed using the log-rank test. Log-rank *p* values and hazard ratios are shown.

We also analyzed the patients stratified according to molecular subtypes (also based on the expression of ER, progesterone receptor (PR), and Her2), LN status, and tumor grade. For miR-181b, there was a significant improvement in overall survival associated with lower levels of miR-181b when all breast cancer patients were included (*n* = 1262, *p* = 0.00029; Figure 8A). Interestingly, upon stratification, there was a significant improvement in overall survival with lower levels of miR-181b expression in luminal B patients (*n* = 433, *p* = 0.0058; Figure 8B), patients with grade II (*n* = 383, *p* = 0.034; Figure 8C) and with grade III (*n* = 620, *p* = 0.019; Figure 8D), and LN-negative patients (*n* = 672, *p* = 0.0035; Figure 8E).

**Figure 9:**
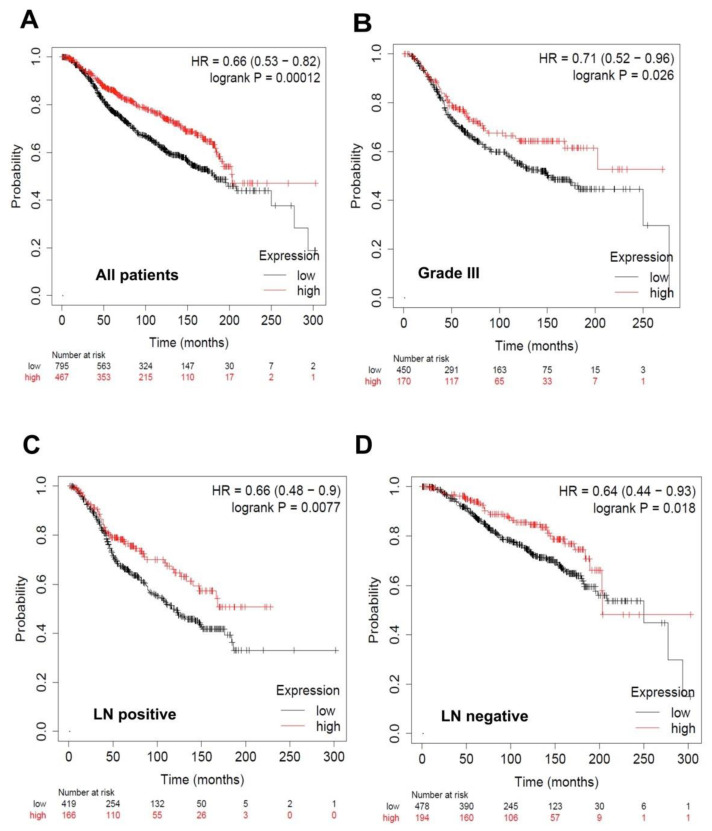
Overall survival curves according to miR-1 expression in breast cancer patients. Low miR-1 expression correlated with worse outcome. Kaplan-Meier curves are plotted for the expression of miR-1 in (**A**) all patients (*n* = 1262), (**B**) patients with grade III (*n* = 620), (**C**) LN-positive patients (*n* = 585), and (**D**) LN-negative (*n* = 672). Curves were analyzed using the log-rank test. Log-rank *p* values and hazard ratios are shown.

For miR-1, a significant improvement in overall survival was observed with higher levels of miR-1 when all breast cancer patients were employed in the analysis (*n* = 1262, *p* = 0.00012; Figure 9A). According to the molecular subtypes, there was no preference in the miR-1 expression level. However, there was a significant increase in overall survival, with higher levels of miR-1 expression in patients with grade III (*n* = 620, *p* = 0.026; Figure 9B) and both lymph node (LN)-positive patients (*n* = 585, *p* = 0.0077, Figure 9C) and LN-negative (*n* = 672, *p* = 0.018; Figure 9D).

For miR-200b and miR-200c, the overall survival of all patients was not significantly changed with the miR-200b expression level. According to the molecular subtypes, there was no change in overall survival as well, except in patients with triple-negative breast cancer, which showed an improved survival with lower levels of miR-200b (*n* = 223, *p* = 0.034; Figure S2A). Apart from miR-200b, there was a significant improvement in the overall survival associated with lower levels of miR-200c when all breast cancer patients were employed in the analysis (*n* = 1262, *p* = 0.00017; Figure S2B). Upon stratification, there was a significant increase in the overall survival, with lower levels of miR-200c expression in luminal B patients (*n* = 433, *p* = 0.025, Figure S2C), patients with grade II (*n* = 383, *p* < 0.0001; Figure S2D), and LN-positive patients (*n* = 585, *p* < 0.0001; Figure S2E). Though being downregulated in the IBC cohort, low miR-200c expression has been associated with better survival in PR^−^ but not in PR^+^ breast cancer [30]. Moreover, circulating miR-200c was upregulated in relapsed breast cancer patients, and low levels have been associated with better distant metastasis-free survival in studied breast cancer patients, and the same findings were observed for miR-200b expression in the same cohort [31]. Taken together, this suggests that miR-200b and miR-200c expression in IBC patients require more comprehensive studies focusing on survival analysis.

For miR-203a, there was a significant improvement in overall survival associated with lower levels of miR-203a when all breast cancer patients were employed in the analysis (*n* = 1262, *p* = 0.027; Figure S3A). Upon stratification, there was no available data for luminal A and luminal B subtypes and patients with different tumor grades. Nonetheless, overall survival was significantly improved, with lower levels of miR-203a in triple-negative breast cancer (*n* = 97, *p* = 0.008; Figure S3B) and LN-negative patients (*n* = 272, *p* = 0.037, Figure S3C).

## 4. Discussion

A large body of evidence indicates that dysregulated expression of miRNAs is associated with disease pathogenesis, including breast cancer [32]. In this study, we demonstrated an upregulation of 10 miRNAs and 18 downregulated miRNAs in tumors of IBC vs. non-IBC. Further, we validated a set of miRNAs that had dysregulated expression, namely miR-181b-5p, miR-200b-3p, miR-200c-3p, miR-203a-3p, and miR-1-3p, and determined their diagnostic accuracy to discriminate IBC from non-IBC patients.

A previous study showed that out of 30 miRNAs that were differentially expressed between breast cancer and normal tissue samples, only 6 had a significant differential expression with a reduction in miR-205 and miR-29b expression levels in IBC vs. non-IBC [10], in accordance with our array findings. However, it should be noted that their study did not enroll a cohort of early stage breast cancer patients, on the contrary to our study. Three previous studies identified a different miRNA profile in IBC. A study by Van der Auwera et al. demonstrated a significant overexpression of miR-335, miR-337-5p, miR-451, miR-486-3p, miR-520a-5p, and miR-548d-5p, whereas miR-15a, miR-24, miR-29a, miR-30b, miR-320, miR-342-5p, and miR-432-3p were significantly downregulated in IBC vs. non-IBC [9]. Another study conducted by Lerebours et al. indicated that 13 miRNAs were differentially expressed and identified a 5-miRNA signature, including miR-421, miR-486, miR-503, miR-720, and miR-1303, with an 89% overall accuracy for IBC patients [17]. Lastly, Maltseva et al. identified a 31-miRNA expression pattern distinct from those profiled in previous studies with a predictive 4-miRNA signature (let7a, miR-582-5p, miR-591, and miR-16-2-3p), which is associated with the p53 encoding gene TP53 mutational status in IBC [16]. The discrepancy in the miRNA profile between these three studies and our array results may originate from the differences in study design, clinic-pathological data (specifically grading and stage IV involvement) of the enrolled patients, and methodology used.

It has been described that miRNAs have the potential to modulate the expression (up- or downregulation) of genes that are involved in a specific metabolic, developmental, or functional process [33]. The KEGG analysis showed that most enriched pathways of the target genes of both up- or downregulated miRNAs were associated with cancer, pathways in cancer, ECM-receptor interaction, and focal adhesion. In agreement, some genomic studies prove that different miRNA genes are located in genomic regions associated with cancer [34]. It is well known that miRNAs regulate a wide range of signaling pathways associated with cancer, but their mechanism is not well understood. The identification of hub genes allowed us to observe that these genes are implicated in a different important process. The hub genes in module 1 are implicated in ubiquitin-mediated proteolysis and the Hedgehog signaling pathway, where both pathways have been proposed as therapeutic targets in different types of tumors [35,36]. Hub genes in module 2 were implicated in endocytosis and cholesterol metabolism. Endocytosis regulates cell adhesion and migration, important steps for metastatic cells [37]. Cholesterol-derived metabolites play important and complex roles in inducing cancer progression and suppressing immune responses, and cholesterol is linked to the aggressiveness of IBC [38,39]. Hub genes in module 3 were mainly associated with neuroactive ligand-receptor interaction and synapsis, chemokine, and cAMP signaling. Some studies demonstrated that the nervous system plays an important role in the progression of glioma and the metastasis of breast cancer cells to the brain [40,41]. Several cytokines and chemokines trigger signaling cascades that activate the nuclear factor-kappa B (NFκB) transcription factor, which is a master regulator of cancer-related immune responses, and this pathway has an especially important implication in IBC [42,43]. Interestingly, hub genes in module 4 were implicated in different types of cancer as well as signaling pathways associated with cancer, including proteoglycans, and human papillomavirus infection. This conforms with our previous study reporting that triple-negative IBC tumors overexpress the heparan sulfate proteoglycan Syndecan-1 [43], the substrate whose functional activity is affected by the heparanase enzyme, which is also overexpressed in tumors of triple-negative IBC [44]. Further, we and others have previously shown that IBC tumors confer multiple viral DNA prevalence [45] and that viral DNA is significantly associated with the triple-negative IBC, the most aggressive phenotype [46]. In accordance with our findings, among the identified hub genes, phosphatidylinositol-4,5-bisphosphate 3-kinase catalytic subunit alpha (PIK3CA), fibroblast growth factor receptor (FGFR), ErbB receptor tyrosine kinase ERBB3 and ERBB4, mammalian target of rapamycin (mTOR), and the ER gene ESR1 have been reported to be either somatic mutated or have frequently altered expression in IBC tumors [47,48]. In the context of signaling pathways, IBC tumors exhibit an aberrant activation of the human epidermal growth factor receptor pathway via overexpression of EGFR, the RAS-MAPK pathway, the PI3K/AKT pathway, and the Src pathway, as well as overexpression of angiogenesis-related genes [39]. Taken together, the hub genes, which are targets of the differentially expressed miRNAs, are involved in important biological processes mainly related to miRNAs, proteoglycans, pathogen infection, signaling pathways, and factors related to the initiation and progression of cancer. This interconnected network of DEGs may uncover the molecular mechanisms underlying the unique biology and pathogenesis of IBC.

Our miRNA PCR array and qPCR data unveiled an overexpression of miR-181c-5p and miR-181b-5p, respectively. This is consistent with a report showing that miR-181c is elevated in IBC relative to adjacent non-neoplastic tissues and that its expression was accompanied by tumor growth via direct targeting of phosphatase and tensin homolog (PTEN) [49]. Furthermore, another study demonstrated that the expression levels of the miR-181 family members are elevated in mammospheres relative to cells grown under two-dimensional (2-D) conditions, concomitantly with reduced expression of its target gene Ataxia telangiectasia mutated (ATM) [50]. Interestingly, miR-181 acts as a downstream target for activin and transforming growth factor-β (TGF-β), promoting breast cancer cell migration and invasion [51]. Of note, 4/17 IBC cases and 1/18 non-IBC case did not display miR-181b-5p expression. Collectively, considering these previous studies and our array as well as qPCR data, it suggests that the miR-181 family members miR-181b, miR-181c, and miR-181d may represent a unique elevated miRNA signature in IBC patients.

The miR-200 family serves as tumor suppressors in a wide range of diseases and tumor entities, including breast cancer [32,52]. Numerous studies reported relevant roles for miR-200b and miR-200c in the inhibition of breast cancer initiation and progression of cancer via suppression of pivotal oncogenic functions and mechanisms, namely the EMT program, metastasis, cancer stem cell phenotype, and chemoresistance [52,53,54,55,56,57]. Apart from the tumor-suppressive role of the miR-200 family, other studies proposed that overexpression of miR-200s promotes the proliferative potential and metastatic capacity of transformed mammary epithelial cells [58]. Another study revealed that elevated miR-200s family levels are associated with increased risk of metastasis in breast cancer and promotes metastatic colonization in mouse models through direct targeting of sec23a [59]. Further, miR-200-overexpressing mouse breast cancer cell lines surprisingly exhibit lung and liver macroscopic metastases via reduced ZEB2 and elevated E-cadherin expression inducing MET [60]. Consistent with our findings, clinical tissues from patients with triple-negative breast carcinoma overexpress miR-200b in comparison to normal tissue samples [61]. This can explain the high expression of miR-200b-3p and miR-200c-3p in tumors of IBC and non-IBC and that their expression was reduced in IBC relative to non-IBC tumors. Interestingly, the accuracy of discrimination IBC from non-IBC was improved upon the combinations of three miRNAs (miR-181b-5p, miR-200b-3p, and miR-200c-3p), with an AUC of 0.897 (sensitivity 84.6%, and specificity 80%), compared to the corresponding individual miRNAs.

Being a direct target, an inverse association of ZEB2 with miR-200b-3p was observed in the whole breast cancer patients without stratification into IBC and non-IBC. This negative correlation may be noted if a larger number of IBC and non-IBC cases are enrolled. ZEB2 is a transcription factor, responsible for the suppression of the epithelial marker and adhesion molecule E-cadherin [28,62,63,64]. However, the high expression of the tumor-promoting E-cadherin in IBC was demonstrated, whereby inducing IBC cell clustering, and mediating the formation of the lymphovascular emboli [65,66]. A study by Ye and coworkers [66] presented evidence that overexpression of E-cadherin in IBC is driven by altered protein trafficking and demonstrated that the human xenograft models of IBC, MARY-X and MARY-X spheroids, displayed downregulated E-cadherin mRNA levels, whereas they overexpressed its protein levels in comparison to E-cadherin-positive human breast carcinoma cells. This could be further supported by the partial existence of epithelial traits, namely a hybrid epithelial/mesenchymal phenotype, as a prerequisite for cluster formation and metastasis in IBC as previously reported [67].

Another important finding in our study is the reduction or loss of miR-203a-3p expression in IBC tumors. This could be driven by epigenetic hypermethylation-mediated silencing of miR-203, resulting in enhanced tumor growth and invasive capacity in malignant breast cancer as previously reported [68]. This is in accordance with different studies, which uncovered that miR-203 directly targets its upstream regulator ZEB2, suppressing EMT in lung adenocarcinoma [69], nasopharyngeal carcinoma [57], prostate cancer [70], and clear cell renal cell carcinoma [71]. In contrast, another mechanism reported for miR-203 regulation is sponging via the competing endogenous RNA (ceRNA) long non-coding RNA (lncRNA) UCA1, which resulted in an upregulated ZEB2-mediated EMT pathway in gastric carcinoma [72]. Several studies have reported downregulation of miR-203 in triple-negative breast [73,74], ovarian [75], colorectal [76], nasopharyngeal [77], and prostate cancers [78,79]. Moreover, its reduced expression is associated with advanced stages, LN metastasis, and poor survival in breast [73], ovarian [75], and colorectal cancers [76]. Furthermore, its overexpression restrains proliferation, migration, and invasion of different cancer cell types [73,74,75,76]. Overexpression/ectopic expression of miR-203 was found to suppress EMT reversing into MET, which is associated with reduced breast cancer stem cell (CSC) properties via targeting of the stemness master regulator ΔNp63 (p63 that lacks the N-terminal transactivation domain) [80]. However, it should be noted that miR-203 has been shown to act as an oncogene in the ER+ MCF-7 breast cancer cells [81], and in ovarian cancer [82], suggesting cell/tumor entity-, and oncogenic state-context-dependent functions. To our knowledge, this is the first study reporting a downregulation of miR-203a-3p, which along with miR-200b-3p and miR-200c-3p may serve as further mechanistic clues for the observed elevated ZEB2 expression levels in IBC patients.

Although no significant difference was identified for altered expression of miR-1-3p in IBC relative to non-IBC tumors, we found a trend for significance for expression loss of miR-1-3p in IBC vs. non-IBC. This is in agreement with previous studies providing in vitro and in vivo evidence that miR-1 functions as a tumor suppressor and its reduced expression is associated with a short overall survival in breast cancer. This is evident as miR-1 is downregulated in breast cancer tissues when compared to normal tissues and re-expression of miR-1 suppresses tumor cell growth, migration, and metastasis and promotes apoptosis in vitro and in vivo via targeting of K-RAS and human metastasis-associated lung adenocarcinoma transcript 1 (MALAT1) [83]. Further, miR-1 has been found to retard proliferation and potentiate apoptosis, and it regulates EMT-related genes in breast CSCs [84]. In contrast, the lncRNA MALAT1 has been found to serve as a ceRNA of the cell division cycle 42 (cdc42) 3′UTR, resulting in enhanced migration and invasion of breast cancer cells by binding miR-1 competitively [85]. Another study suggests that MALAT1 regulates the miR-1/Slug axis through a reciprocal negative regulation in triple-negative breast cancer [86]. In contrast, a study by Minemura et al. reported that 20% of breast carcinoma cases are positive for miR-1, whereas non-neoplastic mammary glands did not express miR-1 as determined by in situ hybridization (ISH), and that miR-1 expression is associated with ER status, PR status, tumor stage, tumor grade, and distant and LN metastasis [87]. This discordance can be caused by the methodology used; ISH vs. qPCR, or by the controls used for normalization; normal healthy mammary tissues (mammoplasty) vs. adjacent non-neoplastic breast tissues.

Finally, the prognostic value of the identified miRNAs has been shown based on both ER and Her2 expression status or according to molecular subtypes, LN status, and tumor grade. Overall, the prognostic impact of the miRNA subset identified to be dysregulated in IBC in our study may be an indicator of the particularly bad outcome of this aggressive breast cancer entity. For example, elevated miR-181b expression is associated with poor prognosis in breast cancer patients with a different classification. Triple-negative breast cancer displaying low levels of miR-200b is associated with an improved survival. This is challenged by the tumor suppression function of miR-200b. Moreover, a study showed that triple-negative breast cancer patients had no expression preference for miR-200b [88]. This may be explained by the fact that triple-negative breast cancer is the most aggressive group of breast cancer, and miR-200b is highly expressed in basal-like metastatic cancer cells [58]. Further, another study by Ye and colleagues emphasized a prognostic advantage for miR-200b expression and that reduced expression levels of miR-200b are associated with poor outcomes of patients with breast cancer [88,89]. In agreement, downregulated miR-200b is associated with poor outcome in breast cancer patients with ER-negative status. Though being downregulated in the IBC cohort, low miR-200c expression has been associated with better survival in PR^-^ but not in PR^+^ breast cancer [30]. Moreover, circulating miR200c was upregulated in relapsed breast cancer patients, and low levels have been associated with better distant metastasis-free survival in the studied breast cancer patients, and the same findings were observed for miR-200b expression in the same cohort [31]. In contrast, KM plotter analysis showed that downregulated miR-200c correlates with worse survival in breast cancer patients with both ER-positive and -negative and Her2-negative status. Taken together, this suggests that miR-200b and miR-200c expression in IBC patients require more comprehensive studies focusing on survival analysis in the context of ER and Her2 expression status.

The few survival studies and low patient numbers for miR-203a in KM datasets hinders comprehensive conclusion; nonetheless, a recent study has suggested no survival advantage for different levels of miR-203a [90]. Different studies reported a significant association of reduced miR-1 with prognosis of triple-negative breast cancer [91] and with a poor overall survival rate in breast cancer [83]. However, miR-1 low levels correlate with improved overall survival in breast cancer patients with ER-positive and Her2-negative status.

One possible caveat that could be associated with this study is the quantification of the expression levels of dysregulated miRNAs in total carcinoma tissue lysates of IBC and non-IBC, which may overlap with miRNAs derived from the infiltrated immune cells, particularly macrophages, as we and others have shown that IBC tumors are known to be a highly infiltrated with CD163+ M2-type tumor-associated macrophages [44]. For example, while miR-200b expression was downregulated in our triple-negative breast cancer cell lines compared to the ER+ luminal cell line MCF-7, it was expressed at a higher level in the IBC cell line SUM-149 compared to the non-IBC triple-negative breast cancer cell line MDA-MB-231. However, in patient tumors, at least a contribution of macrophages can very likely be ruled out as Cobos Jiménez and coworkers [92] showed a specific signature of miRNAs in monocytes/polarized macrophages using next-generation sequencing. Interestingly, according to their study findings, none of our validated miRNAs, namely miR-200b-3p, miR-200c-3p, miR-203a-3p, and miR-1-3p, were expressed in monocytes/polarized macrophages. Only miR-181b-5p was downregulated in M2a macrophages, which is contrary to our finding in IBC tumors, where miR-181b-5p was upregulated. Together, this strongly suggests that the identified five miRNAs in IBC tumors are not confined to the infiltrated macrophages.

## 5. Conclusions

In conclusion, we identified in this study a panel of differentially expressed miRNAs and detected an upregulation of one the downstream targets, *ZEB2*, in carcinoma tissues of IBC vs. non-IBC. The identified miRNA signature, namely miR-181b-5p, miR-200b-3p, miR-200c-3p, and miR-203a-3p, can be used individually to discriminate IBC from non-IBC patients. More importantly, the combination of the three miRNAs (miR-181b-5p, miR-200b-3p, and miR-200c-3p) robustly improved the accuracy of that discrimination. Further, we proposed that miR-181b-5p, miR-200b-3p, miR-200c-3p, and miR-1-3p may have the potential to act as prognostic markers for IBC patients, given the relevance of ER and Her2 expression status. Moreover, in silico and target prediction analysis revealed that the dysregulated markers regulate numerous processes relevant to IBC progression. However, our findings should be verified in a prospective large cohort of IBC patients and the possibility of using them as non-invasive blood circulating biomarkers and their theranostic potential should be further examined. Additionally, a prospective study is needed to elucidate comprehensively the predicted gene targets and their associated signaling pathways for the identified miRNAs.

## Figures and Tables

**Table 1:** Clinic-pathological characteristics of inflammatory breast cancer (IBC) and non-IBC patients.

Characteristic	IBC (*n* = 17)	Non-IBC (*n* = 18)	*p* Value
Age (years)			
Range	33–82	32–73	0.806 ^a^
Mean ± SEM	54.4 ± 3.2	53.3 ± 2.7	
Tumor size (cm), *n* (%)			
≤4	6 (35)	11(61)	0.11 ^b^
>4	9 (53)	5 (28)	
NA	2 (12)	2 (11)	
Lymph node status, *n* (%)			
<4	5 (29)	12 (67)	0.02 * ^b^
≥4	10 (59)	4 (22)	
NA	2 (12)	2 (11)	
Tumor grade, *n* (%)			
Grade I	0 (0)	1 (5)	0.14 ^b^
Grade II	11 (65)	12 (67)	
Grade III	4 (23)	2 (11)	
NA	2 (12)	3 (17)	
Lymphovascular invasion, *n* (%)			
Negative	4 (23)	11 (61)	0.005 * ^b^
Positive	11 (65)	3 (17)	
NA	2 (12)	4 (22)	
ER, *n* (%)			
Negative	8 (47)	9 (50)	0.85 ^b^
Positive NA	7 (41) 2 (12)	9 (50) 0 (0)	
PR, *n* (%)			
Negative	10 (59)	10 (56)	0.83 ^b^
Positive NA	7 (41) 0 (0)	6 (33) 2 (11)	
Her2, *n* (%)			
Negative	10 (59)	12 (66)	0.28 ^b^
Positive NA	6 (35) 1 (6)	3 (17) 3 (17)	

Data are expressed as mean ± SEM, NA Data not available; * Significant *p* value calculated by ^a^ Student’s *t*-test or ^b^ Pearson Chi-Square. ER, estrogen receptor; PR, progesterone receptor; Her2, human epidermal growth factor receptor 2.

**Table 2:** Fold change of differentially expressed (microRNAs) miRNAs in IBC compared to non-IBC.

miRNAs	Fold Change (log2)
**Upregulated in IBC**	
let-7b-5p	0.82
miR-100-5p	0.82
miR-140-5p	0.82
miR-181b-5p	0.83
miR-181c-5p	0.82
miR-181d-5p	0.83
miR-199a-3p	0.84
miR-222-3p	0.83
miR-328-3p	0.81
miR-495-3p	0.82
**Downregulated in IBC**	
miR-1-3p	−3.18
miR-107	−1.18
miR-129-5p	−1.15
miR-141-3p	−1.17
miR-145-5p	−1.16
miR-148a-3p	−1.15
miR-15b-5p	−1.18
miR-182-5p	−1.17
miR-200b-3p	−1.16
miR-200c-3p	−1.17
miR-203a-3p	−2.17
miR-205-5p	−2.18
miR-206-5p	−1.22
miR-20b-5p	−1.17
miR-210-3p	−1.17
miR-29b-3p	−1.16
miR-485-5p	−1.16
miR-96-5p	−1.17

**Table 3:** Gene ontology (GO) analysis of differentially expressed genes (DEGs) of dysregulated miRNAs.

miRNA Regulation	Category	Term	Count	%	*p*-Value
Upregulated	GOTERM_ BP_FAT	GO: 0006355 regulation of transcription	102	2.8	3.00 × 10^−11^
	GOTERM_ BP_FAT	GO:0006351 transcription	80	2.2	5.00 × 10^−08^
	GOTERM_ BP_FAT	GO: 0051252 regulation of RNA metabolic process	67	1.8	2.90 × 10^−06^
	GOTERM_ BP_FAT	GO: 0006355 regulation of transcription, DNA-dependent	63	1.7	2.30 × 10^−05^
	GOTERM_ BP_FAT	GO: 0050808 synapse organization	9	0.2	3.60 × 10^−05^
	GOTERM_ CC_FAT	GO: 0043228 non-membrane-bounded organelle	57	1.6	3.30 × 10^−02^
	GOTERM_ CC_FAT	GO: 0043232 intracellular non-membrane-bounded organelle	57	1.6	3.30 × 10^−02^
	GOTERM_ CC_FAT	GO: 0045177 apical part of cell	8	0.2	3.50 × 10^−02^
	GOTERM_ CC_FAT	GO: 0031982 membrane-bounded vesicle	17	0.5	3.50 × 10^−02^
	GOTERM_ CC_FAT	GO: 0008023 transcription elongation factor complex	3	0.1	3.70 × 10^−02^
	GOTERM_ MF_FAT	GO: 0046872 metal ion binding	133	3.6	1.90 × 10^−07^
	GOTERM_ MF_FAT	GO: 0043167 ion binding	135	3.7	2.50 × 10^−07^
	GOTERM_ MF_FAT	GO: 0043169 cation binding	133	3.6	3.50 × 10^−07^
	GOTERM_ MF_FAT	GO: 0008270 zinc ion binding	81	2.2	1.00 × 10^−05^
	GOTERM_ MF_FAT	GO: 0046914 transition metal ion binding	93	2.5	1.20 × 10^−05^
Downregulated	GOTERM_ BP_FAT	GO: 0007507 heart development	36	0.3	1.80 × 10^−08^
	GOTERM_ BP_FAT	GO: 0010629 negative regulation of gene expression	62	0.6	1.90 × 10^−08^
	GOTERM_ BP_FAT	GO: 0006468 protein amino acid phosphorylation	75	0.7	2.40 × 10^−08^
	GOTERM_ BP_FAT	GO: 0006351 transcription	176	1.6	5.60 × 10^−08^
	GOTERM_ BP_FAT	GO: 0045892 negative regulation of transcription	56	0.5	1.30 × 10^−07^
	GOTERM_ CC_FAT	GO: 0031981 nuclear lumen	116	1	9.80 × 10^−07^
	GOTERM_ CC_FAT	GO: 0005794 Golgi apparatus	76	0.7	6.10 × 10^−06^
	GOTERM_ CC_FAT	GO: 0005581 collagen	11	0.1	6.80 × 10^−06^
	GOTERM_ CC_FAT	GO: 0005583 fibrillar collagen	7	0.1	1.30 × 10^−05^
	GOTERM_ CC_FAT	GO: 0012505 endomembrane system	68	0.6	2.20 × 10^−05^
	GOTERM_ MF_FAT	GO: 0004672 protein kinase activity	68	0.6	2.60 × 10^−07^
	GOTERM_ MF_FAT	GO: 0030528 transcription regulator activity	135	1.2	2.90 × 10^−07^
	GOTERM_ MF_FAT	GO: 0004674 protein serine/threonine kinase activity	50	0.4	4.90 × 10^−06^
	GOTERM_ MF_FAT	GO: 0003700 transcription factor activity	90	0.8	1.20 × 10^−05^
	GOTERM_ MF_FAT	GO: 0016564 transcription repressor activity	39	0.3	1.70 × 10^−05^

BP, biological process; MF, molecular function; cellular component, CC.

**Table 4:** Kyoto Encyclopedia of Genes and Genomes (KEGG) enrichment pathway analysis of DEGs of dysregulated miRNAs.

miRNA Regulation	Category	Term	Count	%	*p*-Value
Upregulated	KEGG_ PATHWAY	hsa04512: ECM-receptor interaction	7	0.2	6.50 × 10^−03^
	KEGG_ PATHWAY	hsa05200: Pathways in cancer	14	0.4	1.30 × 10^−02^
	KEGG_ PATHWAY	hsa05218: Melanoma	6	0.2	1.30 × 10^−02^
	KEGG_ PATHWAY	hsa05214: Glioma	5	0.1	3.70 × 10^−02^
	KEGG_ PATHWAY	hsa04115: p53 signaling pathway	5	0.1	4.70 × 10^−02^
Downregulated	KEGG_ PATHWAY	hsa04360: Axon guidance	24	0.2	9.00 × 10^−07^
	KEGG_ PATHWAY	hsa05200: Pathways in cancer	40	0.4	8.70 × 10^−06^
	KEGG_ PATHWAY	hsa04010: MAPK signaling pathway	34	0.3	2.00 × 10^−05^
	KEGG_ PATHWAY	hsa04510: Focal adhesion	28	0.3	2.80 × 10^−05^
	KEGG_ PATHWAY	hsa05215: Prostate cancer	17	0.2	3.90 × 10^−05^

**Table 5:** miR-1-3p expression detection in carcinoma tissues of IBC as compared to non-IBC.

	miR-1-3p Expression		Pearson-Chi Square (*p*)
	Negative (N)	Positive (N)	
Non-IBC	4	14	0.06
IBC	9	8	

**Table 6:** The prognostic value of the expression of up- and downregulated miRNAs in breast cancer patients stratified by estrogen receptor (ER) and human epidermal growth factor receptor 2 (Her2) expression status.

**miRNA**		**ER**				**Her2**			
		**Positive (*n* = 979)**		**Negative (*n* = 283)**		**Positive (*n* = 157)**		**Negative (*n* = 1105)**	
		HR 95% CI	*p* Value	HR 95% CI	*p* Value	HR 95% CI	*p* Value	HR 95% CI	*p* Value
**Upregulated**	let-7b-5p	0.72 (0.56–0.92)	**0.0083**	1.38 (0.94–2.02)	0.096	0.56 (0.35–0.91)	**0.018**	0.75 (0.6–0.93)	**0.01**
	miR-100-5p	0.65 (0.52–0.8)	**0.00025**	0.78 (0.52–1.19)	0.25	0.53 (0.32–0.87)	**0.011**	0.7 (0.57–0.88)	**0.0015**
	miR-140-5p	0.66 (0.53–0.84)	**0.00048**	0.67 (0.46–0.98)	**0.039**	0.68 (0.4–1.14)	0.14	0.67 (0.54–0.84)	**0.00043**
	miR-181b-5p	1.56 (1.21–2.01)	**0.00046**	1.45 (0.97–2.16)	0.066	0.76 (0.45–1.15)	0.16	1.48 (1.17–1.86)	**0.00092**
	miR-181c-5p	0.57 (0.45–0.72)	**2.5 × 10^−06^**	1.24 (0.85–1.82)	0.26	0.68 (0.42–1.11)	0.12	0.66 (0.53–0.82)	**0.00014**
	miR-181d-5p	0.7 (0.55–0.88)	**0.0022**	1.78 (1.09–2.92)	**0.02**	0.63 (0.46–0.86)	**0.0039**	0.77 (0.62–0.95)	**0.015**
	miR-199a-3p	0.66 (0.51–0.84)	**0.00067**	1.29 (0.85–1.94)	0.23	1.3 (0.81–2.08)	0.28	0.66 (0.53–0.82)	**0.00014**
	miR-222-3p	1.44 (1.09–1.91)	**0.011**	1.35 (0.93–1.96)	0.11	1.55 (0.96–2.51)	0.073	1.38 (1.06–1.79)	**0.016**
	miR-328-3p	0.76 (0.57–1.02)	0.065	0.69 (0.45–1.07)	0.098	0.6 (0.37–0.98)	**0.039**	0.8 (0.64–1.01)	0.056
	miR-495-3p	0.66 (0.52–0.84)	**0.00079**	0.84 (0.58–1.21)	0.35	0.68 (0.42–1.09)	0.11	0.66 (0.53–0.84)	**0.00043**
**Downregulated**	miR-1-3p	0.64 (0.49–0.85)	**0.0015**	0.73 (0.46–1.16)	0.18	0.62 (0.38–1.02)	0.059	0.64 (0.49–0.83)	**0.00077**
	miR-107	1.24 (0.97–1.58)	0.091	0.79 (0.52–1.19)	0.25	0.75 (0.47–1.22)	0.25	1.22 (0.97–1.54)	0.092
	miR-129-5p	1.45 (1.13–1.86)	**0.0037**	0.83 (0.56–1.25)	0.37	0.68 (0.41–1.14)	0.14	1.36 (1.07–1.71)	**0.011**
	miR-141-3p	1.53 (1.21–1.93)	**0.00029**	1.47 (1–2.17)	**0.049**	1.63 (0.98–2.69)	0.055	1.45 (1.17–1.8)	**0.00071**
	miR-145-5p	0.81 (0.63–1.03)	0.084	1.15 (0.79–1.68)	0.45	1.32 (0.78–2.22)	0.3	0.79 (0.63–0.98)	**0.033**
	miR-148a-3p	0.61 (0.48–0.78)	**4.3 × 10^−05^**	1.51 (0.98–2.31)	0.058	0.72 (0.43–1.2)	0.2	0.65 (0.52–0.82)	**0.00028**
	miR-15b-5p	1.26 (0.98–1.62)	0.075	0.82 (0.54–1.24)	0.34	0.64 (0.4–1.04)	0.067	1.3 (1.04–1.64)	**0.024**
	miR-182-5p	0.87 (0.67-1.13)	0.31	1.48 (1–2.19)	**0.049**	0.62 (0.37–0.96)	**0.033**	0.84 (0.65–1.08)	0.18
	miR-200b-3p	0.9 (0.71–1.13)	0.36	1.72 (1.12–2.66)	**0.013**	1.4 (0.83–2.36)	0.2	1.15 (0.91–1.46)	0.25
	miR-200c-3p	1.5 (1.18–1.9)	**0.00083**	1.49 (1.01–2.2)	**0.045**	1.48 (0.92–2.4)	0.1	1.53 (1.21–1.92)	**0.00028**
	miR-203a-3p	NO DATA FOUND							
	miR-205-5p	0.66 (0.52–0.84)	**0.00055**	1.4 (0.94–2.07)	0.094	1.47 (0.89–2.44)	0.13	0.72 (0.58–0.9)	**0.0036**
	miR-206-5p	1.31 (1–1.71)	0.051	1.3 (0.84–2)	0.23	1.36 (0.85–2.19)	0.2	1.34 (1.04–1.72)	**0.022**
	miR-20b-5p	0.8 (0.61–1.05)	0.11	1.17 (0.8–1.71)	0.41	1.46 (0.89–2.38)	0.13	1.11 (0.87–1.41)	0.42
	miR-210-3p	1.62 (1.26–2.09)	**0.00014**	1.97 (1.36–2.85)	**0.00025**	1.39 (0.86–2.26)	0.18	1.6 (1.26–2.02)	**9.3** **×** **10^−05^**
	miR-29b-3p	0.66 (0.52–0.85)	**0.00091**	0.63 (0.43–0.91)	**0.014**	1.26 (0.76–2.09)	0.37	0.6 (0.47–0.76)	**2.1 × 10^−05^**
	miR-485-5p	0.62 (0.48–0.8)	**0.00015**	1.19 (0.78–1.84)	0.42	0.77 (0.47–1.28)	0.32	0.61 (0.49–0.76)	**1.2 × 10^−05^**
	miR-96-5p	0.75 (0.59–0.96)	**0.02**	1.2 (0.79–1.81)	0.39	0.6 (0.37–0.98)	**0.038**	0.76 (0.61–0.95)	**0.018**

Log-rank *p*-values and hazard ratios (HRs; 95 % confidence interval in parentheses) are shown. Bold typing of *p*-values indicates a significant association (*p* < 0.05). Black typing is associated with better overall survival, while red typing is associated with worse overall survival. Gene chip data were used for this analysis.

## Supplementary Materials

The following are available online at https://www.mdpi.com/2218-273X/10/7/1059/s1, **Figure S1**. The network of DEGs protein interactors. String database output depicting functional and physical interactors of all DEGs of differentially regulated miRNAs. **Figure S2**. Kaplan-Meier analysis of overall survival for expression of miR-200b and miR-200c in breast cancer patients. Kaplan-Meier curves are plotted for expression of miR-200b in triple-negative breast cancer patients (*n* = 223) (**A**) and of miR-200-c (**B**) in all breast cancer patients (*n* = 1262), (**C**) Luminal B patients (*n* = 433), (**D**) patients with grade II (*n* = 383), and (**E**) LN-positive patients (*n* = 585, *p* < 0.0001). Curves were analyzed using the log-rank test. Log-rank *p* values and hazard ratios are shown. **Figure S3**. Kaplan-Meier analysis of overall survival for expression of miR-203a in breast cancer patients. Kaplan-Meier curves are plotted for expression of miR-203a in (**A**) all patients (*n* = 1262), (**B**) Triple-negative breast cancer patients (*n* = 97), and (**C**) LN-negative patients (*n* = 272). Curves were analyzed using the log-rank test. Log-rank *p* values and hazard ratios are shown. **Supplementary Table S1**. Clinic-pathological features of IBC and non-IBC patients enrolled for miRNA PCR array analysis. **Supplementary excel Table 1**. BP, CC, MF and KEEG analysis of target genes of upregulated miRNAs. **Supplementary excel Table 2**. BP, CC, MF and KEEG analysis of target genes of downregulated miRNAs. **Supplementary excel Table 3**. MCODE MODULES Hub genes. **Supplementary excel Table 4**. KEEG pathways of Hub genes.
