# Supplementary material for: Inflammatory Breast Carcinoma: Elevated microRNA miR-181b-5p and Reduced miR-200b-3p, miR-200c-3p, and miR-203a-3p Expression as Potential Biomarkers with Diagnostic Value
Source: Biomolecules. 2020 Jul 16;10(7):1059. doi: 10.3390/biom10071059 (PMC7407124; doi:10.3390/biom10071059)
Supplement: Supplementary file 1 [file biomolecules-10-01059-s001.zip › Table S1.docx]

| Characteristic | IBC (*N* = 9) | Non-IBC (*N* =9) | | *P* value | | |  |
| --- | --- | --- | --- | --- | --- | --- | --- |
| Age (years) |  |  | | 0.97^a^ |  |  |  |
| Range  Mean ± SEM | 33-72  51.5 **±** 4.4 | 30-68  51.7 **±** 3.5 |  |  |  |  |  |
| Tumor size (cm), n (%) |  |  | |  | | |  |
| ≤ 4 | 2 (22.2) | 5 (55.6) | | 0.13ᵇ | | |  |
| > 4 | 6 (66.7) | 3 (33.3) | |  | | |  |
| NA | 1 (11.1) | 1 (11.1) | |  | | |  |
| Lymph node status, n (%) |  |  | |  | | |  |
| < 4 | 3 (33.3) | 5 (55.6) | | 0.34ᵇ | | |  |
| ≥ 4 | 6 (66.7) | 4 (44.4) | |  | | |  |
| Tumor grade, n (%) |  |  | |  | | |  |
| Grade I | 0 (0) | 0 (0) | | 0.31ᵇ | | |  |
| Grade II | 6 (66.7) | 7 (77.8) | |  | | |  |
| Grade III | 3 (33.3) | 1 (11.1) | |  | | |  |
| NA | 0 (0) | 1 (11.1) | |  | | |  |
| Lymphovascular invasion, n (%) |  |  | |  | | |  |
| Negative | 4 (44.4) | 7 (77.8) | | 0.15ᵇ | | |  |
| Positive | 5 (55.6) | 2 (22.2) | |  | | |  |
| ER, n (%) |  |  | |  | | |  |
| Negative | 5 (55.6) | 3 (33.3) | | 0.23^b^ | | |  |
| Positive  NA | 3 (33.3)  1 (11.1) | 6 (66.7)  0 (0) | |  | | |  |
| PR, n (%) |  |  | |  | | |  |
| Negative | 5 (55.6) | 5 (55.6) | | 0.77^b^ | | |  |
| Positive  NA | 4 (44.4)  0 (0) | 3 (33.3)  1 (11.1) | |  | | |  |
| HER-2, n (%) |  |  | |  | | |  |
| Negative | 5 (55.6) | 6 (66.7) | | 0.59^b^ | | |  |
| Positive  NA | 3 (33.3)  1 (11.1) | 2 (22.2)  1 (11.1) | |  | | |  |
|  | | | | | | | |

**Table S1**. Clinic-pathological features of IBC and non-IBC patients enrolled in miRNA PCR array analysis.

Data are expressed as mean ± SEM, NA Data not available

*Significant *P* value calculated by ªStudent’s t-test or ᵇPearson Chi-Square
