# Supplementary figures and images for: Inflammatory Breast Carcinoma: Elevated microRNA miR-181b-5p and Reduced miR-200b-3p, miR-200c-3p, and miR-203a-3p Expression as Potential Biomarkers with Diagnostic Value
Source: Biomolecules. 2020 Jul 16;10(7):1059. doi: 10.3390/biom10071059 (PMC7407124; doi:10.3390/biom10071059)

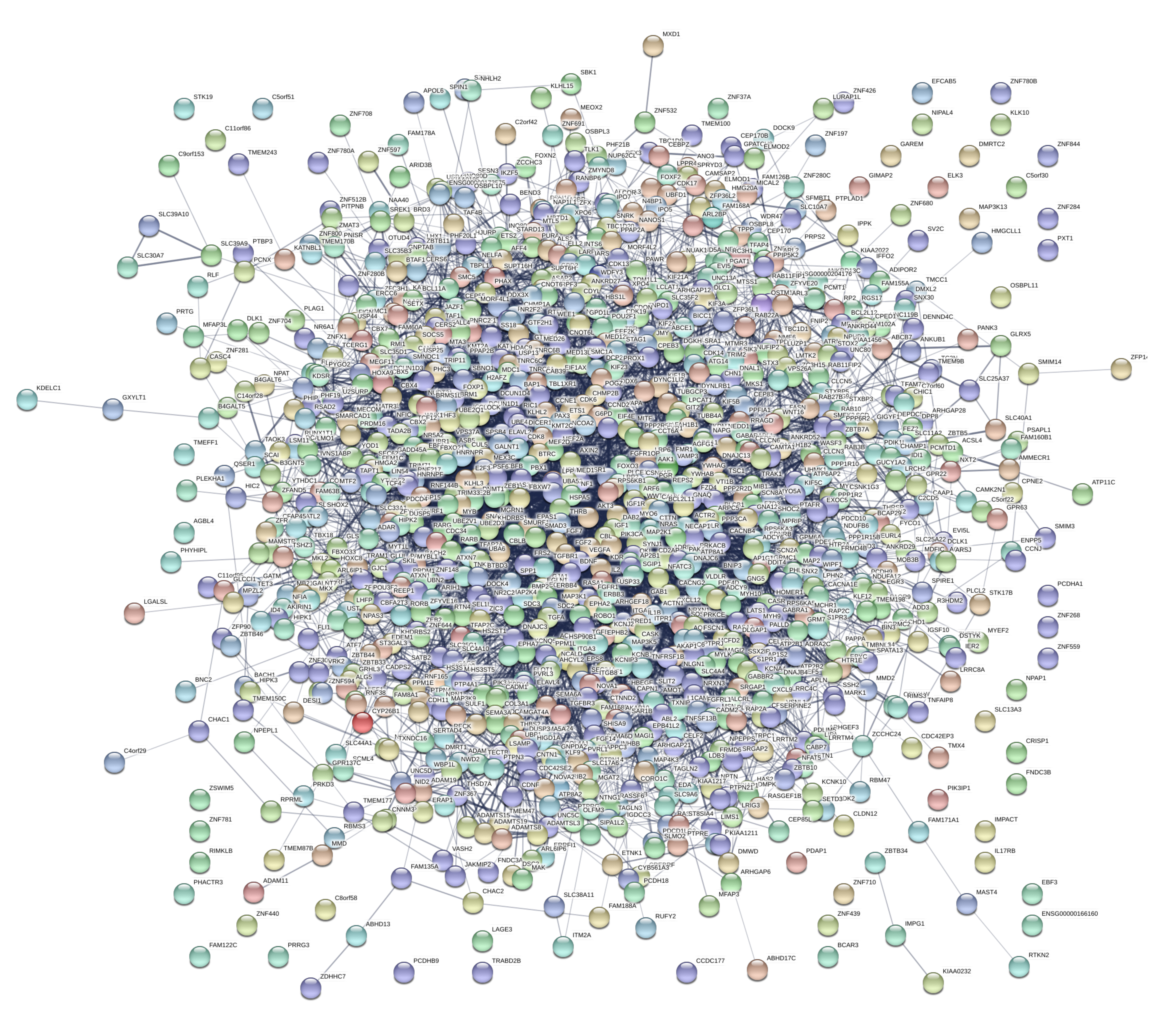

Supplement: Supplementary file 1 [file biomolecules-10-01059-s001.zip › Figure S1]
